# Expression of the Aryl Hydrocarbon Receptor in Growth Plate Cartilage and the Impact of Its Local Modulation on Longitudinal Bone Growth

**DOI:** 10.3390/ijms16048059

**Published:** 2015-04-10

**Authors:** Therése Cedervall, Pia Monica Lind, Lars Sävendahl

**Affiliations:** 1 Department of Women’s and Children’s Health, Pediatric Endocrinology Unit, Astrid Lindgren Children’s Hospital, Q2:08, Karolinska University Hospital Solna, S-171 76 Stockholm, Sweden; E-Mail: lars.savendahl@ki.se; 2 Department of Medical Sciences, Occupational and Environmental Medicine, Uppsala University Hospital, 751 85 Uppsala, Sweden; E-Mail: monica.lind@medsci.uu.se

**Keywords:** AhR (aryl hydrocarbon receptor), epiphyseal growth plate, dioxin, alpha-naphthoflavone, chondrocyte, bone growth

## Abstract

Although dioxin has been reported to impair bone growth in both humans and animals, the underlying mechanisms have not been clarified. We conducted this study to rule out if dioxin may directly target the growth plate, via local modulation of the aryl hydrocarbon receptor **(**AhR). Initial studies in rare tissue samples of the human growth plate confirmed that the AhR protein is widely expressed in growth plate cartilage. To explore the local role of the AhR, mechanistic studies were performed in a well-established model of cultured fetal rat metatarsal bones. The longitudinal growth of these bones was monitored while being exposed to AhR modulators. The AhR agonist, 2,3,7,8-tetrachlorodibenzo-*p*-dioxin, did not affect bone growth at any concentrations tested (1 pM–10 nM). In contrast, the AhR antagonist, alpha-naphthoflavone, suppressed bone growth and increased chondrocyte apoptosis, although only at a high, potentially cytotoxic concentration (50 µM). We conclude that although the AhR is widely expressed in the growth plate, bone growth is not modulated when locally activated, and therefore, dioxin-induced growth failure is likely mediated through systemic rather than local actions.

## 1. Introduction

Dioxins are endocrine disrupting environmental contaminants that induce a broad spectrum of biological responses in wildlife and humans, including growth retardation. The toxic effects of dioxins generally are ascribed to their binding and activation of the aryl hydrocarbon receptor (AhR). The AhR is a ligand-activated transcription factor, and when bound to a ligand, it activates the transcription of numerous genes, including the metabolizing enzyme cytochrome P450 family [1,2].

Bone development is an endocrine-regulated process that occurs in the epiphyseal growth plate where chondrocytes proliferate and form a cartilaginous matrix. Determined by the differentiation of the chondrocytes, three different zones of cells can be distinguished within the growth plate cartilage: the resting, proliferative and hypertrophic zones. The cartilage will function as a template to be replaced later with bone tissue by osteoblasts and osteoclasts, and the balance between proliferation and differentiation of chondrocytes is crucial in bone elongation. Different hormones and growth factors, e.g., estrogen, growth hormone (GH) and insulin-like growth factor-1 (IGF-1), act systemically and/or locally in the cartilage tissue to regulate this process [3,4].

Both epidemiological studies and *in vivo* experimental studies have demonstrated a growth suppressive effect of dioxins [5,6,7,8,9]. Adult, *in utero* and lactational exposure to 2,3,7,8-tetrachlorodibenzo-*p*-dioxin (TCDD) alter bone elongation in rats, with different sensitivities depending on the type of AhR allele that the rat strain possesses [6,7]. Recently, Hattori *et al.* [10] found that treatment of pregnant rats with TCDD (1 µg/kg body weight) reduced the body weight of their fetuses and decreased the levels of systemic GH by more than 50%. The same group also found that pituitary expression of GH and serum levels of IGF-1 were decreased by TCDD in mice [11]. Similarly, Lindén *et al.* [12] found that treating adult rats with a large dose of TCDD (100 µg/kg body weight) decreased serum IGF-1 levels and, interestingly, increased fibroblast growth factor 21 (FGF21) levels, which was not the case in food-restricted controls. FGF21, which modulates glucose and lipid metabolism during fasting, has been shown to prevent the local actions of GH at the growth plate level, thereby inhibiting chondrocyte proliferation and differentiation [13].

We conducted this study to clarify if in addition to systemic effects, the AhR also acts locally in the growth plate chondrocytes to mediate effects on bone elongation. First, we confirmed that the receptor is expressed in the human growth plate to ensure its potential role at this site and the relevance in human susceptibility. The AhR protein was found to be expressed in both boys and girls throughout puberty. Second, we micro-dissected the metatarsal bones from rat fetuses and followed their growth in culture with either the strongest agonist, TCDD, or the best known available antagonist, alpha-naphthoflavone (αNF), for the receptor.

## 2. Results and Discussion

### 2.1. Expression of the AhR in Human Growth Plate Chondrocytes

**Figure 1:**
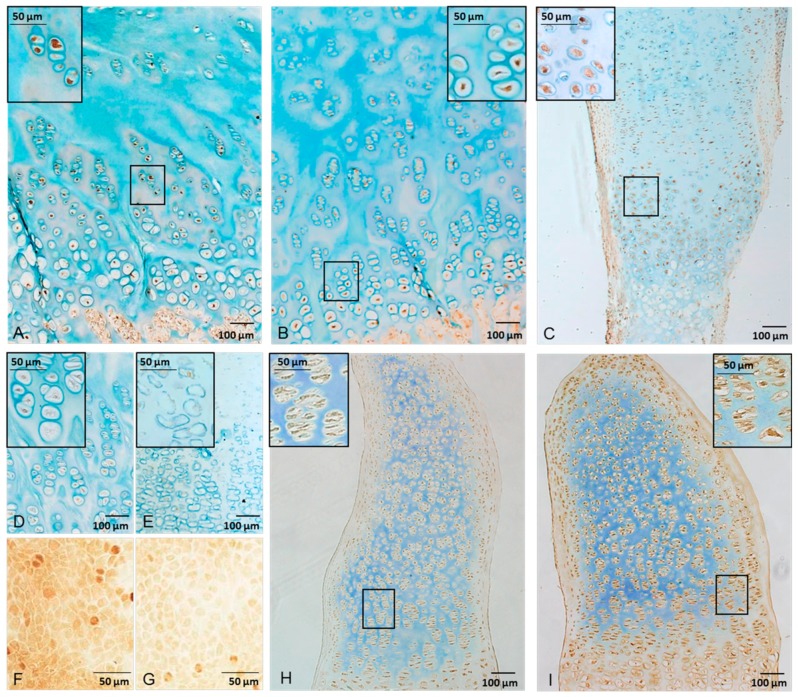
Immunohistochemistry pictures of AhR protein expression in human growth plate tissues from one boy in late puberty, Tanner Stage G5 (**A**), and one girl in early puberty, Tanner Stage B1 (**B**). Chondrocyte expression of the AhR was also confirmed in the fetal rat metatarsal bones used in the culture experiments (**C**). As negative controls for human growth plate (**D**) and rat metatarsal (**E**), tissue was incubated with unspecific IgG instead of primary antibody. The specificity of the AhR (H-211) rabbit polyclonal antibody was validated in HaCaT keratinocytes transfected with either an empty vector expressing the AhR (**F**) or a knockdown vector to express a minimum amount of the protein (**G**). Expression of CYP1A1 in metatarsals after 19 days in culture with either vehicle (**H**) or 10 nM TCDD (**I**).

**Figure 2:**
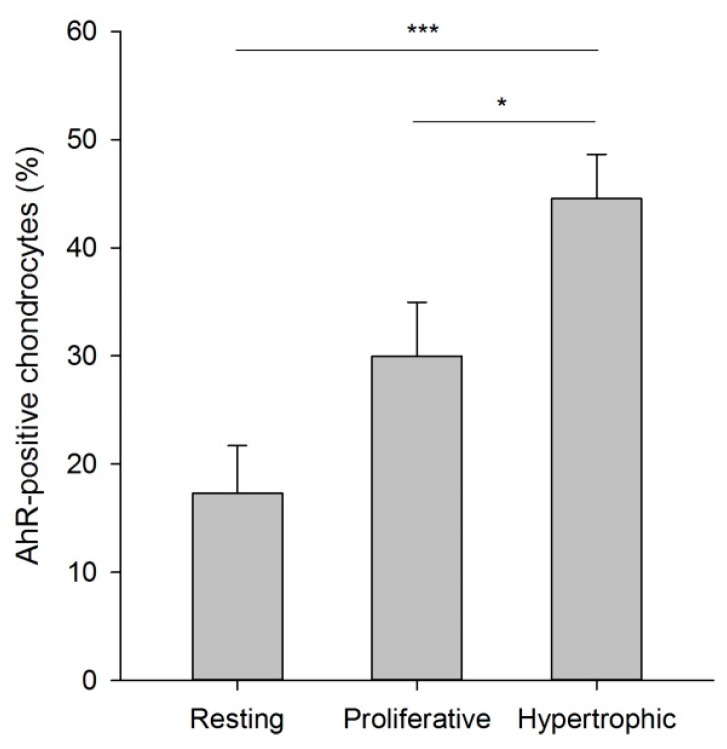
Percentage of AhR-positive chondrocytes in human growth plate tissue, detected by immunohistochemistry. The protein expression was most extensive in hypertrophic chondrocytes. Data are presented as the mean ± SEM, *n* = 7. * *p* < 0.05; *** *p* < 0.001.

Immunohistochemistry studies confirmed that the AhR is widely expressed in the human growth plate cartilage. It was expressed in all samples from boys and girls and throughout puberty (Table 1; Figure 1A,B). The proportion of cells expressing the AhR protein was higher in differentiated chondrocytes with an average of 44.6% ± 4.1% positive cells in the hypertrophic zone compared to 30.0% ± 5.0% (*p* < 0.05) in the proliferative zone and 17.2% ± 4.4% (*p* < 0.001) in the resting zone (Figure 2). The specificity of the AhR (H-211) rabbit polyclonal antibody was verified in AhR knockdown HaCaT keratinocytes, which generated a weak signal compared to empty-vector cells (Figure 1F,G). AhR expression was also confirmed in the metatarsal bones used in the culture experiment (Figure 1C) and quantified to 41% positive cells in the proliferative zone. Our data showing a higher abundance of the AhR protein in more differentiated growth plate chondrocytes are in line with an earlier report in chicks, where a 5.8-fold increase in AhR gene expression was demonstrated in hypertrophic compared to proliferative chondrocytes [14]. Interestingly, we note a tendency towards decreased expression in the resting zone in biopsies collected at later stages of puberty. However, all patients from whom growth plate samples were collected in late puberty were boys. Tissue samples from a larger number of patients could have clarified if the AhR expression changes during pubertal development or if there are any sex differences. Unfortunately, the limited access to human growth plate tissues makes this difficult.

**Table 1:** Descriptive data of the patients from whom tissue samples were collected and the abundance of AhR protein expression in the different growth plate zones.

Patient Information					AhR Positive Chondrocytes (%) ^3^			
#	Diagnosis	Sex ^1^	Age (Years)	Pubertal Stage ^2^	Resting Zone	Proliferative Zone	Hypertrophic Zone	All Zones
1	Constitutional tall stature	F	12	B1	28.7	30.6	50.7	36.6
2	Constitutional tall stature	F	12	B2	34.6	28.8	35.5	33.0
3	Constitutional tall stature	M	13	G3	18.6	11.9	40.3	23.6
4	Leg length difference	M	15	G3-4	5.6	17.2	38.9	20.6
5	Leg length difference	M	14	G4-5	0	46.9	42.6	29.9
6	Klinefelter syndrome	M	12	G5	7.5	16.7	29.4	17.9
7	Leg length difference	M	16	NA	15.3	39.4	52.7	35.8

^1^ Sex: F = female; M = male; ^2^ the pubertal staging of the patients was performed according to the Tanner scale: B = breast development; G = genital development; NA = no available information; ^3^ AhR-positive chondrocytes expressed as the percentage of the total number of chondrocytes in the respective zone.

### 2.2. Effects of TCDD and αNF on Fetal Rat Metatarsal Bone Growth, Apoptosis and Proliferation

**Figure 3:**
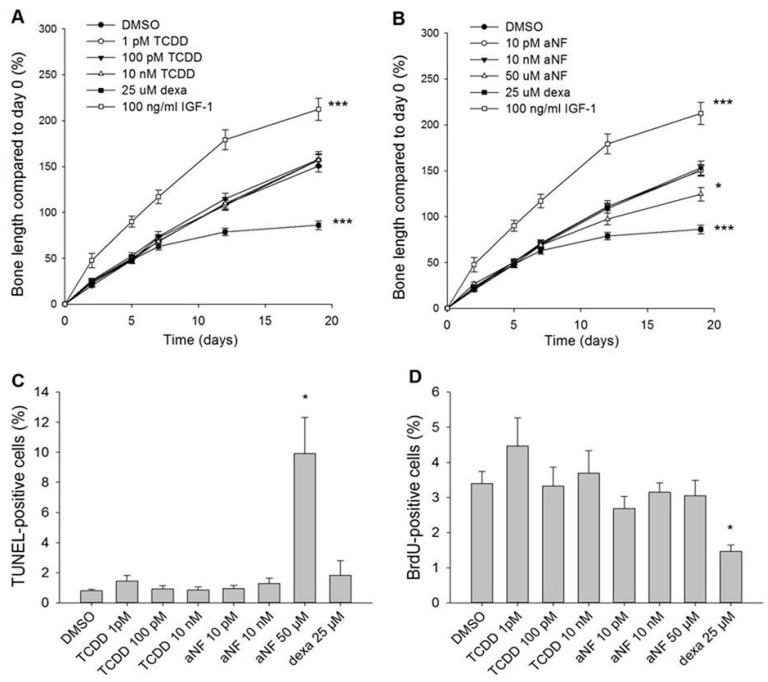
Growth curves of fetal rat metatarsal bones cultured and treated for 19 days with either the agonist TCDD (**A**) or the antagonist αNF (**B**), *n* = 10–12. Percentages of apoptotic chondrocytes detected with TUNEL-staining (**C**) and of proliferating chondrocytes detected with BrdU-incorporation analysis (**D**) in the cultured bones, *n* = 3–6. Data are presented as the mean ± SEM. * *p* < 0.05; *** *p* < 0.001.

The AhR antagonist αNF retarded bone growth at the highest concentration, 50 µM, decreasing it by 17.4% ± 7.5% compared to the vehicle control DMSO after 19 days in culture (Figure 3B; *p* < 0.05). At lower concentrations (10 pM and 10 nM), αNF did not affect the bone growth. The number of apoptotic and proliferating growth plate chondrocytes was analyzed at the end of the culture experiment. Terminal deoxynucleotidyl transferase (TdT)-mediated deoxy-UTP nick end labeling (TUNEL) showed that the only treatment that significantly increased the percentage of apoptotic cells was 50 µM αNF, with 9.9% ± 2.4% positive cells compared to 0.8% ± 0.1% in the vehicle control group (Figure 3C; *p* < 0.05). TCDD did not affect metatarsal bone growth in the tested concentrations, 1 pM–10 nM (Figure 3A). The positive and negative growth modulators, IGF-1 and dexamethasone (dexa), altered the bone growth by +41.1% ± 12.0% or −42.8% ± 4.7%, respectively, compared to the vehicle control (*p* < 0.001). TCDD did not affect the amount of apoptosis in the treated metatarsals, and the percentage of proliferating chondrocytes, measured with bromodeoxyuridine (BrdU)-incorporation, was neither altered by TCDD nor αNF (Figure 3D). Dexa decreased the percentage of BrdU-positive cells to 1.5% ± 0.18% compared to 3.4% ± 0.34% in the vehicle control (*p* < 0.05). To verify that TCDD had induced activation of the receptor in the metatarsals, the expression of CYP1A1 was analyzed with immunohistochemistry. The expression was upregulated by all concentrations of TCDD, with the most intense staining in 10 nM-treated bones (Figure 1H,I).

A high dose of the antagonist αNF resulted in retarded metatarsal growth with 17% and increased the amount of apoptosis. Cytotoxicity of αNF in µM concentrations has been described to be independent of the AhR in AhR-containing and AhR-deficient rat hepatoma cell lines [15]. It is therefore likely that this effect was due to cytotoxic effects rather than through specific blockage of AhR signaling. However, it cannot be excluded that functional AhR-signaling is required to maintain chondrocyte viability. The agonist TCDD did not alter the metatarsal growth in any of the tested concentrations. The concentration range of TCDD was chosen to reflect the environmental exposure to dioxin and is known to be sufficient to activate the receptor in other *in vitro* models [16], and the receptor activation in the treated metatarsals was confirmed with increased CYP1A1 expression. In medaka embryos, 1 h of exposure to 0.05 ppb TCDD was sufficient to cause defects in hypural cartilage development [9]. Chondrocytes derived from articular cartilage in rabbits were reported earlier to be triggered into apoptosis by nM concentrations by both TCDD and polychlorinated polychlorinated 126 [17,18]. It is therefore surprising to us that TCDD exerted no local effect on bone elongation in rat metatarsals. It could be speculated that an unknown factor in serum is needed for the AhR in the rat growth plate to enable effects on cartilage formation.

## 3. Experimental Section

### 3.1. Subjects

Human growth plate tissue samples were obtained from 5 boys and 2 girls subjected to epiphyseal surgery at different stages of puberty in order to arrest further leg growth, due to constitutional tall stature or leg length difference. No underlying disease was present in these patients (except one patient who had Klinefelter syndrome), and we therefore have no reason to suspect any growth plate pathology. The subjects’ gender, age, pubertal stage and diagnoses are presented in Table 1. In connection with this surgery, growth plate biopsies from the proximal tibia and distal femur were sampled using bone marrow biopsy needles (7 gauge; Gallini Medical Products and Services, Modena, Italy). The pubertal staging of the patients was performed by a trained pediatric endocrinologist according to the Tanner scale. The study was preapproved by the local ethics committee at Karolinska University Hospital, Stockholm, Sweden, and informed consent was obtained from the patient and both parents.

### 3.2. Culture of Fetal Rat Metatarsal Bones

The three middle metatarsal bones were dissected from the hind paws of fetal Sprague-Dawley rats (19–20 days post-conception). The bones were cultured 19 days in supplemented phenol red-free DMEM-F12, as previously described [19]. Because photoproducts of the culture medium ingredient tryptophan alter background levels of CYP1A1-activity [20], the medium was protected from UV-radiation by aluminum foil. Treatment with TCDD (Cambridge Isotope Laboratories, Andover, MA, USA), αNF, the positive control IGF-1 or negative control dexa (Sigma-Aldrich, Inc., Steinheim, Germany) was added in the culture media when changed every second or third day. TCDD was handled with special precautionary procedures. Digital images of cultured bones were captured at Days 0, 2, 5, 7, 12 and 19 of the culture using a stereo microscope (Nikon SMZ-U, Nikon, Tokyo, Japan) connected to a digital camera (Hamamatsu C4742–95, Hamamatsu, Hamamatsu City, Japan) and a computer. Images were analyzed using the Image-ProPlus image analysis software (Media Cybernetic, Gleichen, Germany) as previously described [21]. The experiment was repeated twice, 5–6 bones per group. The growth of the metatarsals (10–12 bones in each group) is expressed as the percentage increase of the length compared to the day of dissection (Day 0). The study was approved by the local animal ethics committee at the Karolinska Institute (Stockholm, Sweden).

### 3.3. Immunohistochemistry

Human growth plate samples and metatarsal bones were fixed, embedded in paraffin and sectioned onto objective slides, and immunohistochemistry was performed as previously described [22]. The primary antibodies, rabbit polyclonal anti-AhR (AhR H-211) (Santa Cruz Biotech Inc., Santa Cruz, CA, USA) or rabbit polyclonal anti-CYP1A1 (Bioss, Woburn, MA, USA), were diluted 1:50 or 1:100, respectively, in 3% horse serum in Tris-buffered saline (TBS) and applied overnight at 4 °C. As a negative control, unspecific rabbit IgG (Vector Laboratories, Burlingame, CA, USA) was applied instead of the primary antibody. The secondary antibody, biotinylated polyclonal goat-anti-rabbit obtained from Dako (Glostrup, Denmark), was diluted 1:400 in 1% bovine serum albumin in TBS and applied for 1 h at room temperature. Pictures for manual counting of AhR-positive cells were taken with 100× magnification; the inserts in Figure 1A–E,H,I were taken with 400× magnification, using a Nikon Eclipse E800 microscope (Nikon Corp., Tokyo, Japan) equipped with an Olympus DP70 digital camera (Olympus Sverige AB, Solna, Sweden).

### 3.4. HaCaT-Cells and Immunocytochemistry

To verify the specificity of the AhR (H-211) rabbit polyclonal antibody, immunocytochemistry was performed on AhR knockdown HaCaT keratinocytes, a human epithelial cell line that carries a knock-down vector for the AhR [23]. The HaCaT cells were a gift from Ellen Fritsche, Leibniz Research Institute for Environmental Medicine, Düsseldorf, Germany. The HaCaT cells were cultured in a 37 °C, 5% CO_2_ incubator, in DMEM media containing 10% fetal calf serum, 1% non-essential amino acids, 1% penicillin/streptomycin and 0.6 mg/mL Geneticin^®^ (Life Technologies, Stockholm, Sweden). In a 24-well plate, 50,000 cells/well were plated onto cover slips and cultured in 0.5 mL culture medium for 48 h prior to fixation in 4% formaldehyde in PBS. Immunocytochemistry was performed as the immunohistochemistry described above, with the following exceptions: After blocking of endogenous peroxidase activity, the cell membranes were permeabilized with ice-cold permeabilization buffer (containing 20 mM HEPES, 300 mM sucrose, 30 mM NaCl and 0.8% TritonX-100) for 10 min. The primary antibody was diluted 1:100 and the secondary antibody 1:400 in 3% horse serum in TBS. The cells were mounted with water-soluble mounting medium (Dako) and pictures taken with 400× magnification.

### 3.5. BrdU

BrdU-incorporation analysis of the metatarsals was performed using a cell proliferation kit (RPN20; Amersham Biosciences, Buckinghamshire, UK) as previously described [24]. The level of proliferation is expressed as the percentage of positive cells per total cell number in the bone. For each group, the number of proliferating cells was determined in 3–6 metatarsals.

### 3.6. TUNEL

Apoptotic cells were identified by TUNEL-immunohistochemistry, according to the instructions for the TdT-FragEL™ DNA fragmentation kit (Oncogene Research, Boston, MA, USA) with previously described modifications [24]. The level of apoptosis is expressed as the percentage of positive cells per total cell number in the bone. For each group, the number of apoptotic cells was determined in 4–6 metatarsals.

### 3.7. Statistical Analysis

The statistical differences of AhR expression between different zones in the human growth plate were calculated by one-way analysis of variance (ANOVA) followed by the Student–Newman–Keuls test. The statistical differences in the metatarsal experiments were calculated by one-way ANOVA followed by the Holm–Sidak method, except for TUNEL-analysis, where the samples failed to meet the requirements of normal distribution, and therefore, Kruskal–Wallis one-way ANOVA on ranks (Dunn’s method) was applied. The values are expressed as the mean ± SEM. Significance was considered at *p*-values less than 0.05.

## 4. Conclusions

The AhR is expressed in human growth plate chondrocytes, which suggests a potential physiological role in these cells. *Ex vivo* cultures of fetal rat metatarsal bones confirmed that the AhR antagonist, αNF, has the capacity to impair longitudinal bone growth and induce chondrocyte apoptosis when applied at a high concentration. On the other hand, AhR activation with TCDD did not affect bone growth, suggesting that dioxin-induced growth failure is likely mediated through systemic rather than local actions. Nevertheless, it cannot be excluded that AhR signaling plays a local role in growth plate homeostasis, as the receptor is widely expressed in this cartilage.

## Abbreviations

AhR: aryl hydrocarbon receptor
αNF: alpha-naphthoflavone
TCDD: 2,3,7,8-tetrachlorodibenzo-*p*-dioxin
dexa: dexamethasone
IGF-1: insulin-like growth factor-1
GH: growth hormone
FGF21: fibroblast growth factor 21
TBS: Tris-buffered saline
TUNEL: terminal deoxynucleotidyl transferase (TdT)-mediated deoxy-UTP nick end labeling
BrdU: bromodeoxyuridine
ANOVA: analysis of variance
